# Transvenous phrenic nerve stimulation improves central sleep apnea, sleep quality, and quality of life regardless of prior positive airway pressure treatment

**DOI:** 10.1007/s11325-021-02335-x

**Published:** 2021-03-20

**Authors:** Alan R. Schwartz, Lee R. Goldberg, Scott McKane, Timothy I. Morgenthaler

**Affiliations:** 1 Perelman School of Medicine, University of Pennsylvania, Philadelphia, USA; 2 Universidad Peruana Cayetano Heredia, Lima, Peru; 3 Respicardia, Inc., Minnetonka, MN USA; 4 Mayo Clinic, Rochester, MN USA

**Keywords:** Central sleep apnea, Transvenous phrenic nerve stimulation, Positive airway pressure

## Abstract

**Study objective:**

Positive airway pressure (PAP) therapy for central sleep apnea (CSA) is often poorly tolerated, ineffective, or contraindicated. Transvenous phrenic nerve stimulation (TPNS) offers an alternative, although its impact on previously PAP-treated patients with CSA has not been examined.

**Methods:**

TPNS responses among PAP-naïve and prior PAP-treated patients from the **rem**edē^®^ System Pivotal Trial were assessed. Of 151, 56 (37%) used PAP therapy before enrolling in the trial. Patients were implanted with a TPNS device and randomized to either active or deferred (control) therapy for 6 months before therapy activation. Apnea-hypopnea index (AHI) and patient-reported outcomes (PRO) were assessed at baseline, and 6 and 12 months following active therapy.

**Results:**

Patients had moderate-severe CSA at baseline, which was of greater severity and more symptomatic in the PAP-treated vs. PAP-naïve group (median AHI 52/h vs. 38, central apnea index (CAI) 32/h vs. 18, Epworth Sleepiness Scale 13 vs. 10, fatigue severity scale 5.2 vs. 4.5). Twelve months of TPNS decreased AHI to <20/h and CAI to ≤2/h. Both groups showed reductions in daytime sleepiness and fatigue, improved well-being by patient global assessment, and high therapeutic acceptance with 98% and 94% of PAP-treated and PAP-naïve patients indicating they would undergo the implant again. Stimulation produced discomfort in approximately one-third of patients, yet <5% of prior PAP-treated participants discontinued therapy.

**Conclusion:**

Polysomnographic and clinical responses to TPNS were comparable in PAP-naïve and prior PAP-treated CSA patients. TPNS is a viable therapy across a broad spectrum of CSA patients.

**Trial registration:**

ClinicalTrials.gov Identifier NCT01816776; March 22, 2013

**Supplementary Information:**

The online version contains supplementary material available at 10.1007/s11325-021-02335-x.

## Introduction

Sleep apnea is characterized by recurrent episodes of reduced or absent ventilation with intermittent hypoxemia and recurrent arousals from sleep. This disorder has been associated with significant cardiopulmonary, metabolic, and neurocognitive dysfunction as well as reduced quality of life [1–6]. A loss of neuromotor drive to upper airway and respiratory pump muscles plays a major role in the pathogenesis of obstructive and central sleep apnea (CSA), respectively [7, 8]. Non-invasive ventilation with positive airway pressure (PAP) has long been considered a first-line therapy for moderate to severe central and obstructive sleep apnea [9–11]. Unfortunately, this therapeutic modality is frequently poorly tolerated [12] or ineffective [13]. In one trial, adaptive servo-ventilation (ASV) treatment was associated with increased cardiovascular and all-cause mortality in CSA patients with heart failure and reduced ejection fraction compared to untreated controls [14].

Despite these adverse outcomes, PAP therapy addresses several factors implicated in the pathogenesis of CSA, albeit with varying degrees of efficacy. Beneficial effects of continuous PAP (CPAP) on CSA can be attributed to increases in lung volume, oxygen stores, and residual upper airway obstruction [15, 16]. These effects account for an approximately 50% reduction in the overall apnea hypopnea index (AHI) and concomitant increases in nocturnal oxygenation [10]. CPAP cannot stabilize ventilation completely and suppress residual apneas, which recur following transient post-apneic hyperpnea and hypocapnia. When not contraindicated, non-invasive positive pressure ventilation such as ASV can maintain ventilation during central apneas, thereby preventing post-apneic hyperpnea from occurring [11, 17]. Despite high pressures, however, ASV can fail to capture ventilation through leaky interfaces that are frequently poorly tolerated [18–20].

Unlike PAP modalities, transvenous phrenic nerve stimulation (TPNS) treats CSA by pacing a hemidiaphragm directly, thereby generating negative intrapleural pressure to inflate the lungs. In 2017, the US Food and Drug Administration approved the **rem**edē^®^ System, an implantable TPNS system consisting of a pulse generator, stimulation lead, and optional sensing lead for the treatment of moderate to severe CSA in adults based on demonstrable improvements in CSA, sleep quality, and quality of life [21, 22]. This device can stabilize ventilation and maintain oxygenation without arousing patients from sleep [23, 24]. It incorporates automated algorithms that drive nightly therapeutic adherence and offers an alternative to PAP therapy for CSA while obviating the need for wearing a mask and breathing on positive pressure. TPNS could therefore overcome the physiologic, clinical, and lifestyle challenges of PAP therapy.

PAP-intolerance can reflect differences in patient profiles, preferences, sleep propensity, and sleep disordered breathing symptoms [12], all of which can impact the results of therapeutic trials. For example, hypoglossal nerve stimulation, a therapy designed to treat obstructive sleep apnea, has been trialed exclusively in PAP-intolerant patients, which can potentially limit the generalizability of trial outcomes [25]. In contrast, the **rem**edē^®^ System Pivotal Trial overcame this limitation by enrolling both PAP-naïve and prior PAP-treated CSA patients. Nevertheless, the relative benefits of TPNS in both groups of CSA patients have not yet been elucidated. The present study was designed to determine whether prior PAP treatment could have biased our assessment of TPNS effectiveness in the **rem**edē^®^ System Pivotal Trial. The primary goal of this post hoc analysis was to examine differential effects of TPNS on CSA, sleep architecture, and patient-reported outcomes in the PAP-naïve and PAP-treated patients enrolled in the **rem**edē^®^ System Pivotal Trial.

## Methods

### Patients

All patients included in this analysis had been enrolled in the **rem**edē^®^7 System Pivotal Trial, a randomized controlled parallel design trial examining the effect of TPNS in patients with moderate to severe CSA on sleep disordered breathing, sleep quality, and daytime function [26]. Briefly, patients became eligible for this trial based on a polysomnogram (PSG) showing AHI (the number of apnea and hypopnea events per hour of sleep) ≥20/h with a majority of central apneas, an obstructive apnea index ≤20% of the total AHI, and at least 30 central apneas during the night. Patients were excluded if their health care team anticipated a need for chronic oxygen therapy or mask-based therapy over the ensuing 6 months after therapy initiation. Moreover, patients needed to be medically stable for 30 days prior to all baseline testing including not using any PAP therapy. Patients were not required to have failed or attempted prior non-invasive PAP mask-based therapy.

### Study design

**Fig. 1:**
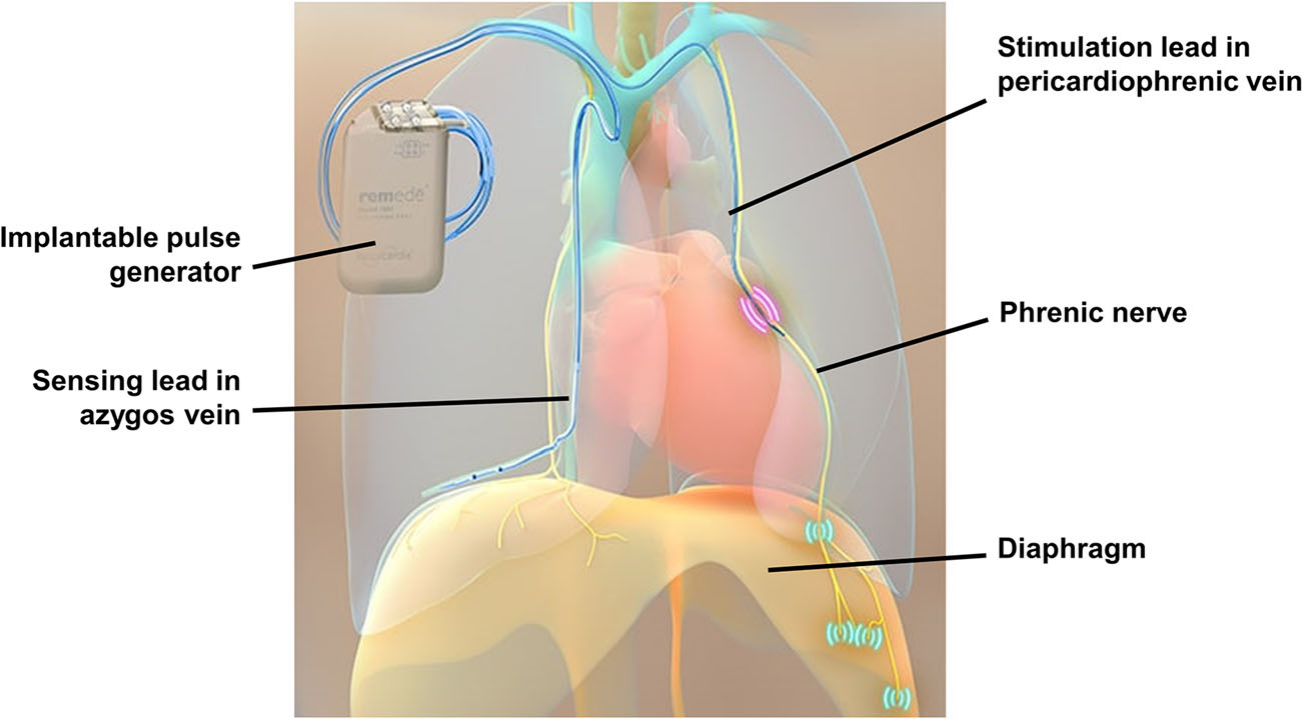
The **rem**edē^®^ System shown with the pulse generator implanted in the right pectoral region, stimulation lead implanted in the left pericardiophrenic vein adjacent to the phrenic nerve, and sensing lead implanted in the azygos vein. The phrenic nerve is stimulated, which travels to and activates the diaphragm to generate a breath

All patients in the pivotal trial were implanted with the TPNS device (**rem**edē^®^ System, Respicardia, Inc., Minnetonka, MN, USA; Fig. 1) and randomized to active TPNS or 6 months deferred therapy before activation. In the present post hoc analysis, results from active and deferred therapy groups were pooled and analyzed based on months of active therapy. Patients were characterized at study enrollment by whether or not they had been treated with PAP prior to enrollment in the trial for this analysis. This history included the type of mask-based therapy used but did not indicate when or why PAP therapy had been stopped. Patients were prohibited from using PAP therapy for ≥30 days before enrollment. All patients then went without any CSA therapy for an additional 1 or 7 months prior to TPNS initiation in the active and control treatment groups of the **rem**edē^®^ System Pivotal Trial, respectively.

### Study procedures

Sleep disordered breathing and sleep architecture parameters were assessed in each patient with in-laboratory attended PSG, which was performed prior to **rem**edē^®^ system implantation and repeated at 6 and 12 months of active therapy. All PSGs were scored by a central core laboratory (Registered Sleepers, Winter Haven, FL, USA) as previously described [21, 26]. Patient-reported outcomes including the Epworth Sleepiness Scale (ESS), Fatigue Severity Scale (FSS), and patient global assessment (PGA) scale were also collected at the visits.

### Outcome variables

Changes in sleep disordered breathing were summarized by the AHI and its components (central, obstructive, and mixed apnea indices and hypopnea index using AASM hypopnea criterion [27]). Sleep quality was represented by sleep stage distributions (non-rapid eye movement N1, N2, and N3, and rapid eye movement sleep as percentages of total sleep time) and arousal index (Arl). Daytime sleepiness was assessed by the ESS, a validated 24-point scale consisting of 8 questions about the patient’s likelihood of falling asleep during sedentary activities with scores above 10 suggesting excessive daytime sleepiness [28]. The FSS measured fatigue with a validated index consisting of 7 items encompassing a scale of 0–7 where ≥4 indicates subjective fatigue. Patients also completed the PGA, a questionnaire that asked “Specifically in reference to your overall health, how do you feel today as compared to how you felt before having your device implanted?,” with seven response levels: markedly improved, moderately improved, mildly improved, no change, slightly worse, moderately worse, or markedly worse. Finally, following 6 months of active therapy, patients were asked “Based on your experience with the **rem**edē^®^ system therapy, would you elect to have this medical device implanted again?”.

### Data analysis

To characterize therapeutic responses to TPNS in the subgroups of patients with and without prior PAP exposure, this post hoc exploratory analysis examined change in measures of sleep disordered breathing, sleep quality, daytime function, and overall satisfaction with therapy. Results at each visit and changes in these metrics from baseline were calculated for continuous endpoints, along with the nominal 2-sided *p*-value from Wilcoxon Signed-Rank test for paired change from baseline within each subgroup after 1 year of active therapy. Nominal *p*-values of < 0.05 were considered statistically significant. Statistical tests were not performed comparing the responses between subgroups since the analysis was not powered to detect such differences. Categorical variables were presented as percentage of patients, and continuous variables were presented as median (25th percentile, 75th percentile) without imputation for missing data. Tolerability of therapy was assessed by review of events related to the delivered therapy through 12 months. SAS version 9.4 (Cary, NC) was used for all analyses.

## Results

### Subject flow, baseline characteristics, and device tolerability

Of the 151 patients enrolled in this trial, 56 (37%) patients used PAP therapy prior to enrolling in the TPNS study. Prior PAP therapy included continuous PAP (*n* = 36, median duration of use 4.5 months [interquartile range 1, 18]), ASV (*n* = 18, median 5 months [1, 12]), bi-level PAP (*n* = 6, median 17 months [4, 48]), and variable bi-level PAP (*n* = 3, median 11 months [3, 50]); 7 patients tried multiple PAP modalities.

Baseline characteristics for PAP-naïve and PAP-treated groups are reported in Table 1. Compared to the PAP-naïve group, the PAP-treated group had a lower prevalence of heart failure (PAP-treated vs. PAP-naïve, 52% vs. 71%) and previous myocardial infarction (14% vs 37%), but had a greater prevalence of depression by history (41% vs. 13%). The PAP-treated group also had a higher baseline AHI, central apnea index (CAI), and ESS, indicating greater CSA severity.

**Table 1:** Baseline characteristics: continuous variables reported as median [first quartile, third quartile] and categorical display percent (*n*)

Baseline characteristics	Prior PAP-treated group (*n* = 56)	PAP-naïve group (*n* = 95)	*p*-value
Male	86% (48)	92% (87)	0.283
Age (years)	67 [60, 75]	64 [59, 73]	0.630
Body mass index (kg/m^2^)	29 [26, 35]	31 [27, 35]	0.402
Heart failure	52% (29)	71% (67)	0.024
Left ventricular ejection fraction	47 [38, 51]	41 [26, 47]	0.008
Atrial fibrillation	38% (21)	45% (43)	0.396
Myocardial infarction	14% (8)	37% (35)	0.003
Concomitant implantable cardiovascular device	29% (16)	51% (48)	0.010
Depression	41% (23)	13% (12)	<0.001
Jaw or neck surgery	9% (5)	1% (1)	0.027
Apnea-hypopnea index (events/hour)	48 [34, 61]	42 [31, 56]	0.076
Central apnea index (events/hour)	30 [18, 45]	20 [14, 36]	0.018
Epworth Sleepiness Scale	12 [6, 16]	8 [5, 13]	0.013

**Fig. 2:**
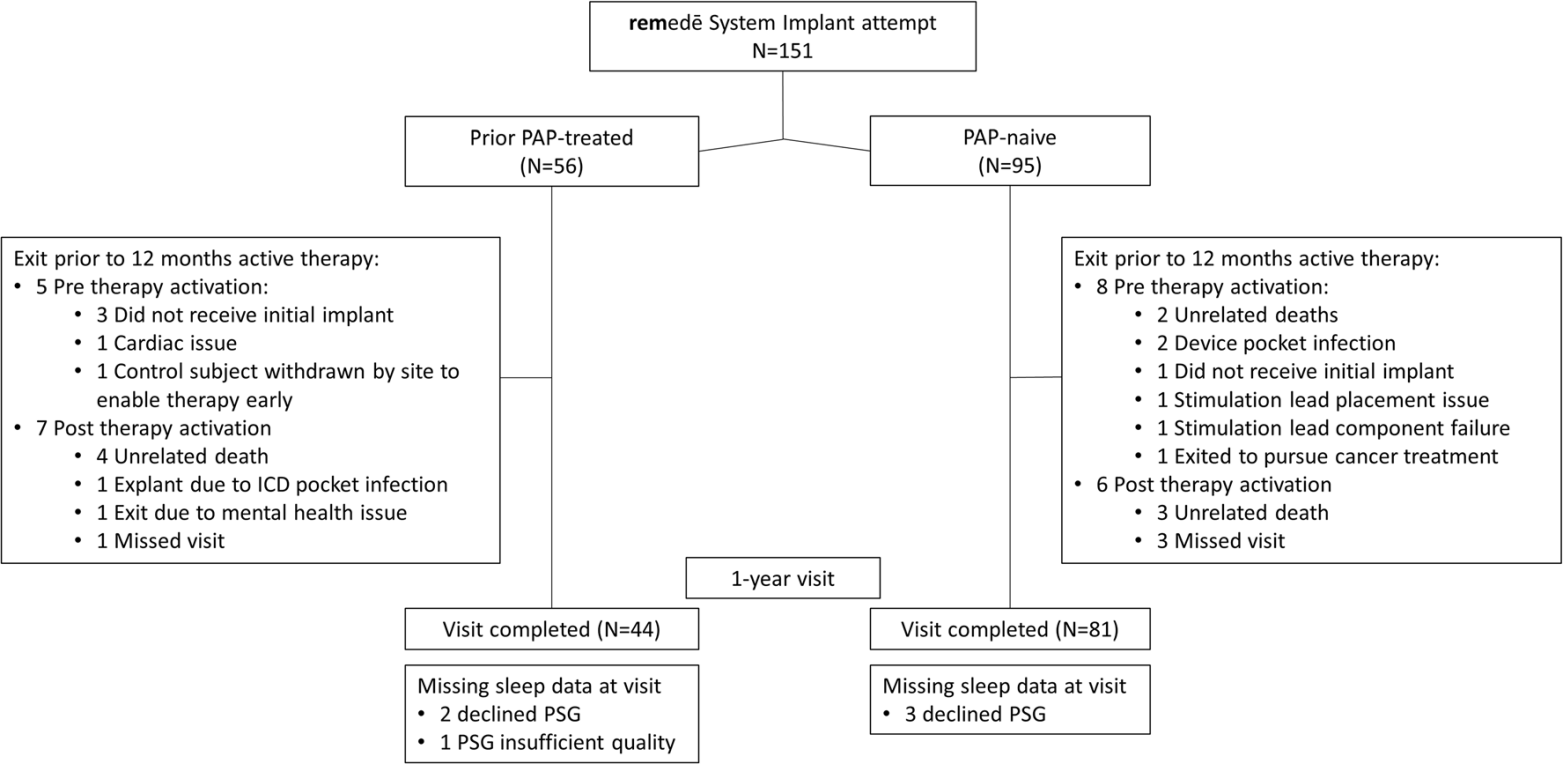
CONSORT Diagram. Patient accountability by prior PAP use subgroup through 1 year of active therapy. PAP positive airway pressure

Patient subgroups derived from the **rem**edē^®^ System Pivotal Trial [21] are illustrated in Fig. 2. In PAP-treated and PAP-naïve groups, 9% and 8% exited the trial prior to therapy activation mainly due to an unsuccessful implant attempt (*n* = 4), device functionality issues (2), or implant site infection (2). Of patients with therapy activated, 14% and 7%, respectively, exited prior to or missed the 1-year post-therapy activation visit. At the 1-year time point, 6% and 4% of patients completing the visit declined PSG or PSG quality was inadequate, respectively.

During the first year of therapy, approximately one-third of PAP-treated patients reported some discomfort during TPNS in the abdominal area, compared to approximately one quarter of the PAP-naïve patients. Two PAP-treated patients (<5%) requested that therapy be stopped for reasons of tolerability (one of whom also had a recurrence of mental health problems). Discomfort resolved after reprogramming the device in all but one of the remaining patients, and this patient chose to continue TPNS anyway.

### Sleep apnea and sleep architecture metrics

**Fig. 3:**
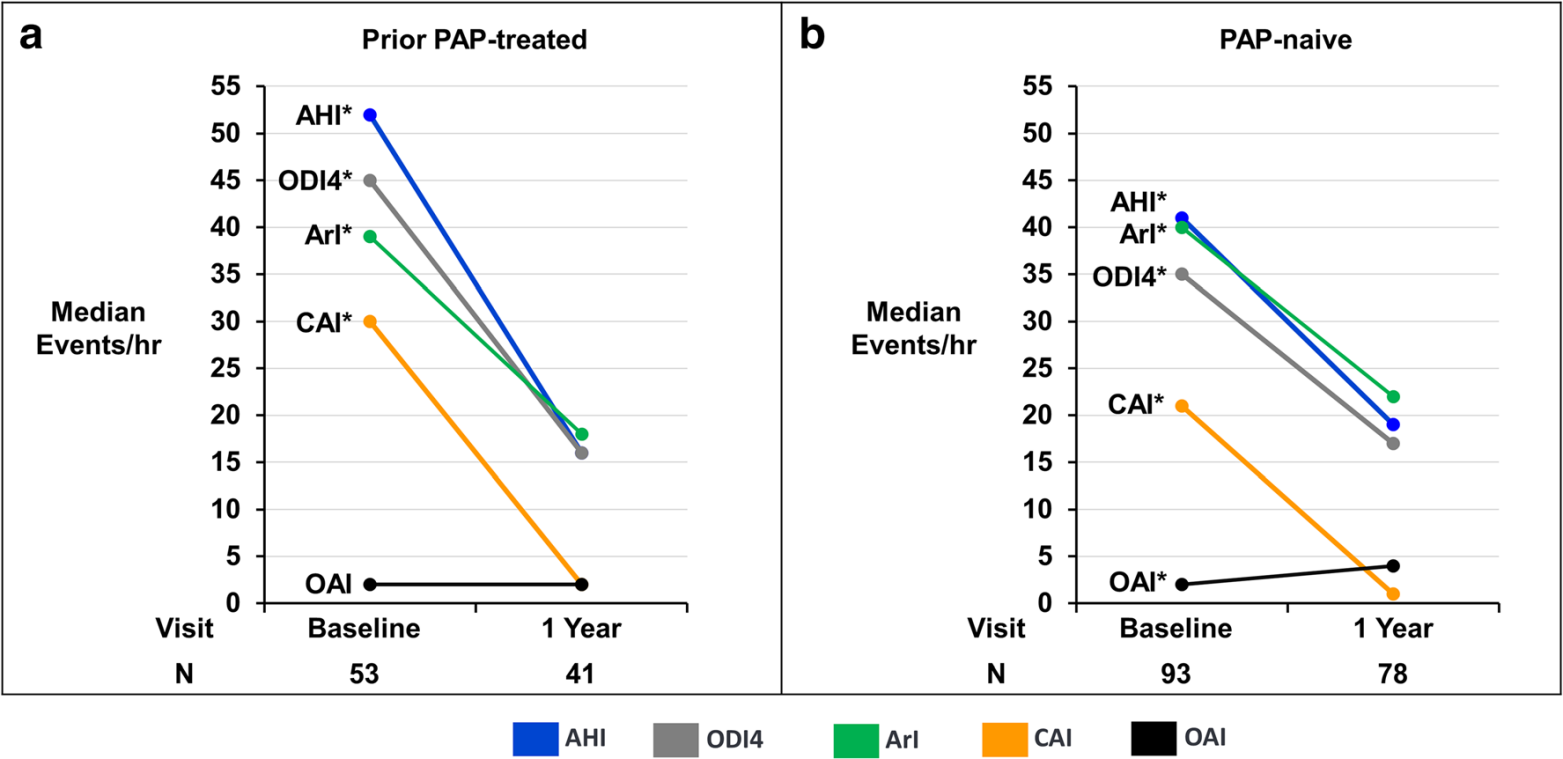
Sleep indices by visit for prior PAP-treated (**a**) and PAP-naïve (**b**) subgroups. Median sleep indices from centrally scored in-laboratory polysomnogram displayed by visit for the prior mask use (left) and no prior mask use subgroups (right). *Paired change from baseline *p* value <.001. AHI apnea-hypopnea index, ArI arousal index, CAI central apnea index, OAI obstructive apnea index, ODI4 4% oxygen desaturation index, PAP positive airway pressure

**Fig. 4:**
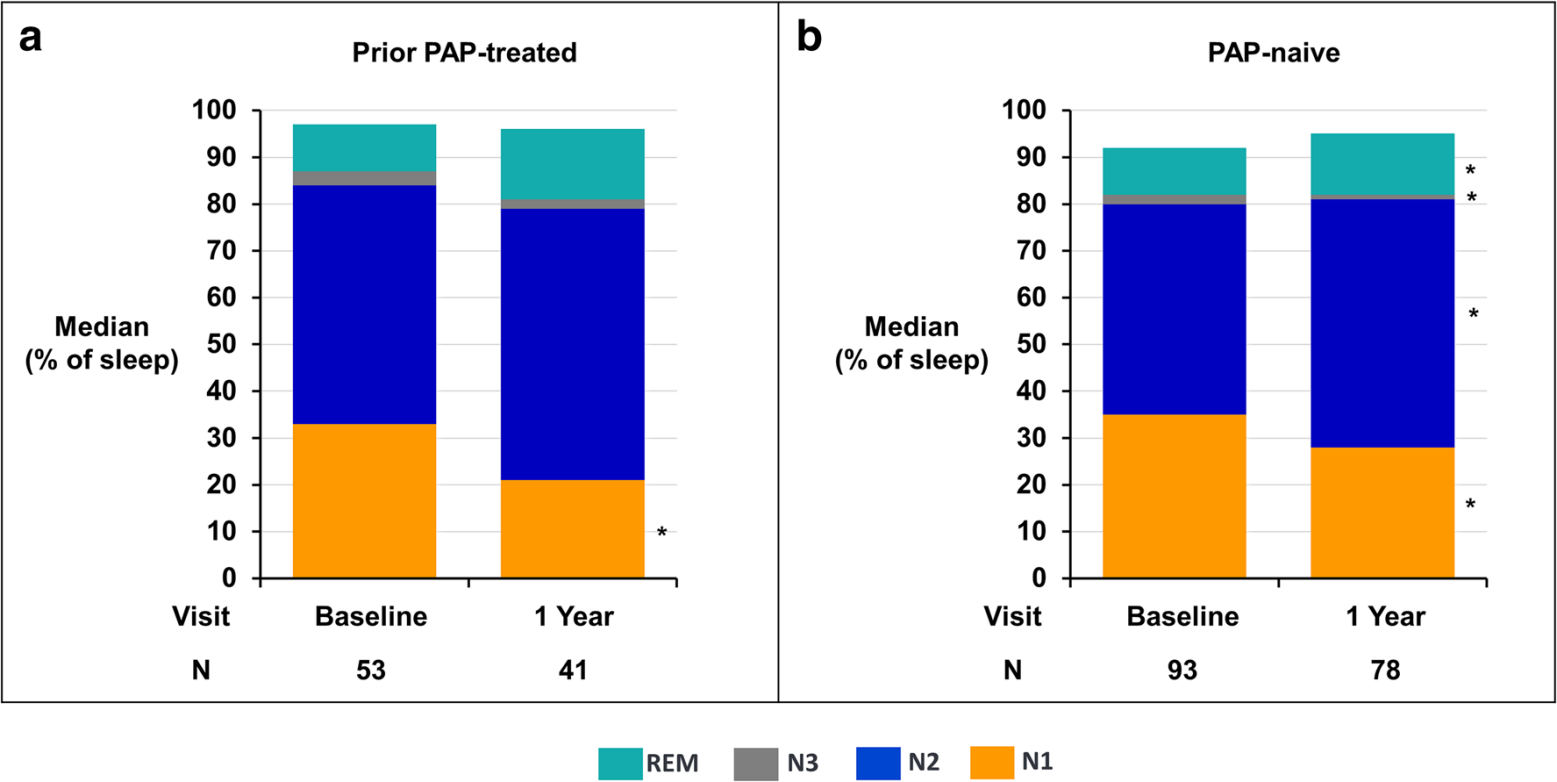
Sleep stages by visit for prior PAP-treated (**a**) and PAP-naïve subgroups (**b**). Median percentage of total sleep time in N1, N2, N3, and REM sleep. The percentage of sleep in light stage sleep (N1) decreased in both subgroups at 1 year. Note that the sum of the medians of the sleep stages does not add to 100%. *Paired change from baseline *p* value < 0.05. PAP positive airway pressure, REM rapid eye movement

Sleep apnea results appear in Fig. 3 and Table 2 for baseline and 1-year after therapy activation in the PAP-treated and PAP-naive subgroups. Clinically and statistically significant decreases in AHI, CAI, 4% oxygen desaturation index, and ArI were observed with TPNS therapy in both groups. Despite higher baseline levels for most sleep indices in the PAP-treated group, both groups demonstrated decreases in these indices to similar levels after 1 year of active therapy with the median AHI falling to <20 events/h and CAI to ≤2 events/h. The obstructive apnea index did not change significantly or increased minimally in the PAP-treated and PAP-naïve groups, respectively. Consistent with the improvements in sleep apnea, sleep architecture improved in both subgroups after 1 year of active therapy with less transitional N1 sleep and greater proportions of N2, N3, and/or rapid eye movement sleep (Fig. 4). Similar improvements in sleep apnea and sleep architecture were observed for both groups at the intermediate 6-month active therapy time point (Online resource Tables 1, 2, 3).

**Table 2:** Polysomnogram and clinical metrics by prior PAP use: median [Q1, Q3]/nominal 2-sided *p*-value from Wilcoxon signed-rank test for paired change from baseline to visit

Endpoint	Prior PAP-treated group			PAP-naïve group		
	Baseline (*n* = 53)	1 year		Baseline (*n* = 93)	1 year	
		Result (*n* = 41)	Paired change from baseline		Result (*n* = 78)	Paired change from baseline
Apnea-hypopnea index (events/hour)	52 [35, 63]	16 [10, 32]	−30 [−45, −7] <.001	41 [31, 56]	19 [9, 34]	−19 [−33, −6] <.001
Central apnea index (events/hour)	30 [17, 47]	2 [0, 6]	−24 [−48, −15] <.001	21 [12, 36]	1 [0, 4]	−17 [−30, −10] <.001
Obstructive apnea index (events/hour)	2 [0, 4]	2 [1, 5]	0 [−1, 3] 0.076	2 [1, 4]	4 [1, 10]	2 [−1, 6] <.001
Mixed apnea index (events/hour)	1 [0, 5]	0 [0, 1]	−1 [−5, −0] <.001	1 [0, 4]	0 [0, 1]	−1 [−3, 0] <.001
Hypopnea index (events/hour)	12 [2, 19]	10 [6, 19]	2 [−7, 7] 0.562	12 [4, 20]	8 [4, 17]	−1 [−7, 7] 0.582
4% oxygen desaturation Index (events/hour)	45 [33, 61]	16 [8, 27]	−26 [−42, −6] <.001	35 [24, 51]	17 [8, 29]	−15 [−29, −3] <.001
Arousal index (events/hour)	39 [30, 54]	18 [14, 26]	−18 [−32, −8] <.001	40 [27, 57]	22 [15, 35]	−12 [−33, −4] <.001
Percent of sleep with O_2_ saturation <90% (%)	12 [4, 27]	5 [1, 13]	−5 [−13, −0] <.001	7 [2, 19]	3 [1, 12]	−3 [−9, 1] <.001
Percent of sleep in N1 (% of sleep)	33 [19, 42]	21 [14, 27]	−7 [−17, 3] 0.023	35 [21, 49]	28 [19, 40]	−4 [−18, 4] 0.002
Percent of sleep in N2 (% of sleep)	51 [42, 58]	58 [48, 65]	4 [−5, 12] 0.116	45 [40, 56]	53 [44, 59]	4 [−3, 13] 0.005
Percent of sleep in N3 (% of sleep)	3 [0, 7]	2 [0, 7]	0 [−1, 1] 0.784	2 [0, 10]	1 [0, 5]	0 [−4, 1] 0.015
Percent of sleep in REM (% of sleep)	10 [5, 16]	15 [6, 21]	4 [−5, 8] 0.251	10 [6, 16]	13 [8, 21]	3 [−2, 11] <.001
Epworth Sleepiness Scale (points)	12 [7, 16]	8 [5, 10]	−3 [−8, 0] <.001	8 [5, 13]	5 [3, 8]	−3 [−6, 0] <.001
Fatigue Severity Scale (points)	5.2 [3.4, 6.2]	4.6 [3.3, 6.0]	−0.2 [−1.4, 0.6] 0.084	4.4 [3.3, 5.7]	3.4 [2.4, 5.1]	−0.5 [−1.6, 0.4] 0.003

Change from baseline in subgroups randomized to active and deferred TPNS therapy (prior to therapy being activated) is displayed in the Online resource Table 4 for the PAP-treated and PAP-naïve subgroups. In both subgroups, active therapy with TPNS produced consistent improvements in polysomnographic metrics compared to little or no change in the inactive control subgroup.

### Patient-reported outcomes

**Fig. 5:**
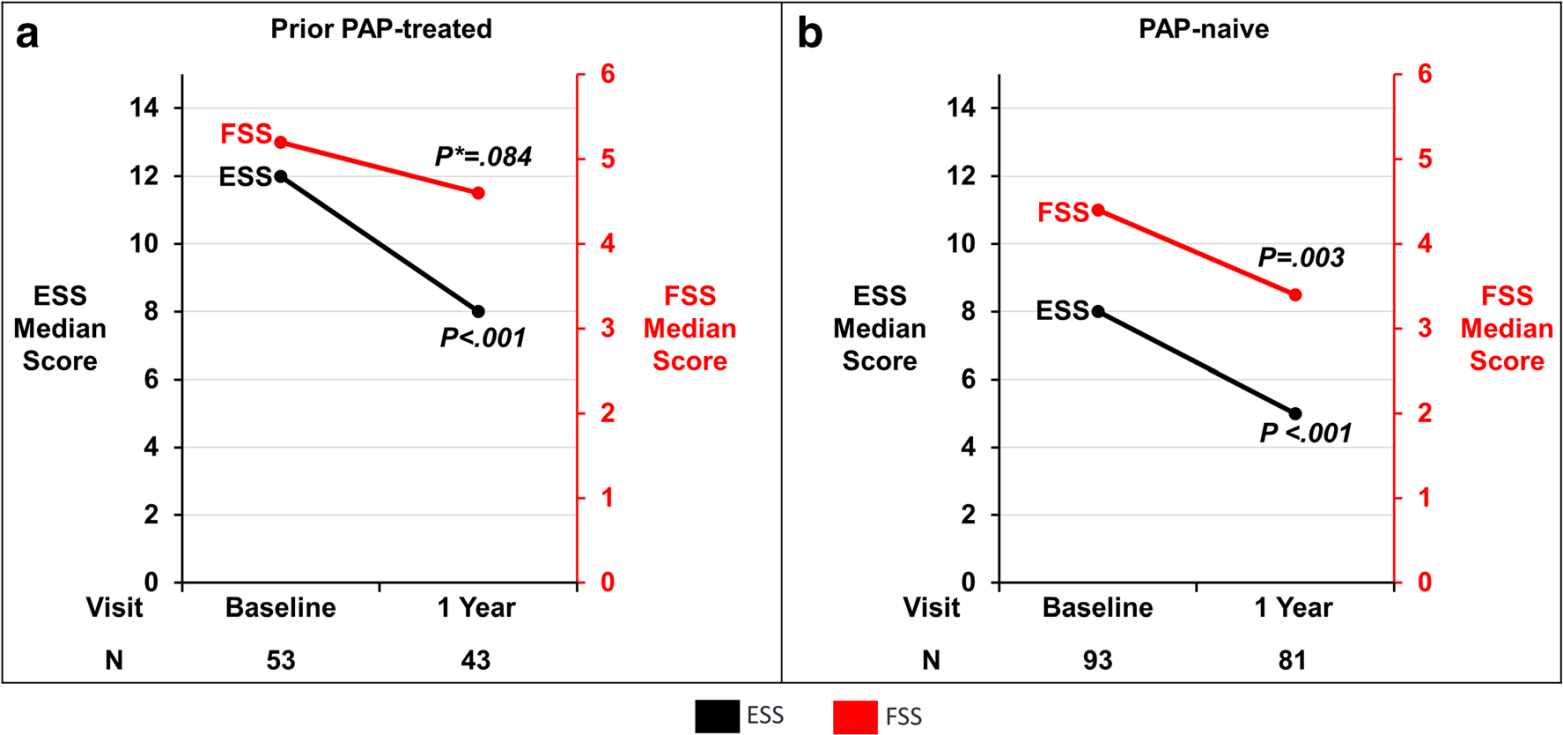
Daytime symptoms for prior PAP-treated (**a**) and PAP-naïve subgroups (**b**). Median scores for ESS and FSS by subgroup at 1 year. The ESS score ranges from 0 to 24 with scores >10 considered excessive daytime sleepiness. The FSS score ranges from 0 to 9 with scores >4 considered excessive fatigue. *Paired change from baseline *p* value. ESS Epworth Sleepiness Scale, FSS Fatigue Severity Scale, PAP positive airway pressure

The median baseline ESS in the PAP-treated group was 12 (interquartile range 7, 16) and the paired change from baseline showed a clinically significant 3 (−8, 0) point improvement after 12 months of TPNS. Despite a lower ESS baseline of 8 (5, 13) in the PAP-naïve group, ESS also improved by 3 (−6, 0) points (Fig. 5 and Table 2). Similarly, the FSS in the PAP-treated group improved by 0.2 (−1.4 to 0.6) and in the PAP-naïve group by 0.5 points (−1.6 to 0.4) at 12 months (Fig. 5 and Table 2). After 1 year of therapy, 84% (36/43) of PAP-treated and 74% (60/81) of the PAP-naïve groups indicated improvement in PGA from baseline. Following 6 months of therapy, the vast majority of patients in the PAP-exposed (98% (46/47)) and in the PAP-naïve (94% (78/95)) groups responded “yes” to indicate that they would undergo the **rem**edē^®^ implantation procedure again. Of note, 1 patient in the PAP-treated group exited prior to the assessment due to a mental health problem, had requested therapy be turned off, and presumably would have responded “No.”

### Matched subgroup analysis

Given differences in baseline characteristics between PAP-naïve and PAP-treated groups, a sensitivity analysis was performed by removing patients with reduced ejection fraction (≤45%) heart failure because one type of PAP therapy (ASV) has since become contraindicated. These restricted PAP-naïve and PAP-treated groups continue to show consistent improvements in sleep apnea, sleep architecture, and daytime function (Table 3). The percentage of patients with improvement in the PGA at 1 year was also similar to the full group (88% (29/33) for PAP-treated and 72% (23/32) for PAP-naïve groups), as was the percentage of patients who, after 6 months of therapy, would have elected to have the device implanted again (100% (34/34) and 97% (31/32)).

**Table 3:** Polysomnogram sleep metrics by prior PAP use—excluding patients with heart failure and ejection fraction ≤ 45%: median [Q1, Q3]/nominal 2-sided *p*-value from Wilcoxon signed-rank test for paired change from baseline to visit

Endpoint	Prior PAP-treated group			PAP-naïve group		
	Baseline (*n* = 35)	1 year		Baseline (*n* = 38)	1 year	
		Result (*n* = 33)	Paired change from baseline		Result (*n* = 32)	Change from baseline
Apnea-hypopnea index (events/hour)	52 [34, 69]	16 [10, 29]	−30 [−45, −7] <.001	38 [27, 57]	17 [7, 32]	−17 [−39, −7] <.001
Central apnea index (events/hour)	32 [17, 50]	3 [1, 7]	−24 [−40, −14] <.001	18 [10, 35]	1 [0, 4]	−16 [−27, −9] <.001
Obstructive apnea index (events/hour)	2 [0, 3]	1 [1, 4]	0 [−1, 3] 0.391	2 [1, 4]	2 [0, 11]	1 [−2, 7] 0.252
Mixed apnea index (events/hour)	1 [0, 3]	0 [0, 1]	−1 [−2, −0] <.001	1 [0, 7]	0 [0, 0]	−1 [−5, 0] <.001
Hypopnea index (events/hour)	12 [2, 19]	11 [5, 19]	2 [−8, 7] 0.569	10 [5, 18]	8 [3, 16]	−2 [−8, 6] 0.446
4% oxygen desaturation index (events/hour)	45 [32, 64]	15 [8, 26]	−28 [−42, −6] <.001	32 [21, 55]	16 [6, 29]	−18 [−32, −3] <.001
Arousal index (events/hour)	36 [24, 48]	18 [14, 26]	−13 [−26, −6] <.001	38 [27, 57]	24 [14, 35]	−10 [−33, −3] <.001
Percent of sleep with oxygen saturation<90% (%)	12 [2, 23]	4 [1, 10]	−7 [−15, −1] <.001	6 [1, 18]	2 [1, 12]	−2 [−8, 0] 0.004
Percent of sleep in N1 (% of sleep)	28 [15, 39]	21 [13, 27]	−7 [−18, 3] 0.025	28 [19, 50]	29 [20, 44]	−3 [−10, 4] 0.176
Percent of sleep in N2 (% of sleep)	52 [44, 63]	58 [48, 66]	4 [−4, 12] 0.143	47 [41, 58]	52 [42, 58]	3 [−4, 12] 0.195
Percent of sleep in N3 (% of sleep)	3 [1, 7]	2 [0, 7]	0 [−1, 2] 0.924	3 [0, 10]	1 [0, 8]	−1 [−4, 0] 0.010
Percent of sleep in REM (% of sleep)	11 [6, 19]	15 [6, 21]	3 [−5, 8] 0.445	10 [6, 13]	13 [6, 21]	2 [−3, 10] 0.083
Epworth Sleepiness Scale (points)	13 [7, 19]	8 [5, 10]	−4 [−9, 0] <.001	10 [5, 13]	6 [4, 8]	−3 [−7, −1] <.001
Fatigue Severity Scale (points)	5.2 [4.3, 6.2]	4.6 [3.3, 5.7]	−0.6 [−1.4, 0.2] 0.020	4.5 [3.3, 5.6]	3.1 [2.2, 4.4]	−0.9 [−1.8, 0.5] 0.009

## Discussion

Among patients with CSA who were enrolled in the **rem**edē^®^ System Pivotal Trial, groups with and without prior PAP therapy both demonstrated clinically and statistically significant improvements in sleep apnea, sleep architecture, and daytime symptoms. Specifically, TPNS proved remarkably effective in eliminating central apneas in both groups and led to substantial improvements in sleep quality, daytime hypersomnolence, and quality of life, despite significant between-group differences in baseline characteristics including CSA severity, sleep symptoms, and cardiovascular co-morbidities. In contrast to PAP-naïve patients, previously treated patients failed PAP therapy, as evidenced by their desire for alternative treatment with TPNS. Physiologic and symptomatic responses to TPNS in this group were similar to those in the PAP-naïve group, suggesting that TPNS can effectively treat a broad spectrum of CSA patients, regardless of PAP treatment status.

Several factors could explain why patients abandoned PAP therapy prior to enrolling in the TPNS trial. First, TPNS offered an alternative to patients with symptomatic sleep apnea because they could not sleep with a mask or device [12]. In fact, tolerability appears to drive greater levels of therapeutic adherence and oxygenation with TPNS than PAP therapy [21, 24]. Second, PAP therapy may not have been efficacious in treating sleep disordered breathing patterns in patients with CSA, even when optimally titrated in the laboratory. Residual sleep disordered breathing would make these patients less likely to respond symptomatically [13, 29]. Third, PAP could have aggravated underlying sleep disturbances, daytime somnolence and depression in the PAP-treated compared to PAP-naïve patients, diminishing the perceived benefits of the prior PAP therapy in this group.

Differences in baseline characteristics between groups may reflect differences in referral pathways from sites participating in the pivotal trial. For example, patients with sleep and psychiatric complaints are likely to present to sleep centers where PAP therapy is readily prescribed for patients with sleep disordered breathing, sleep disturbances, and daytime somnolence [18–20, 30]. In contrast, a greater proportion of the PAP-naïve group had underlying cardiovascular disease, suggesting this group may have been recruited primarily from cardiology rather than sleep clinics. Cardiologists might neither have routinely screened patients for sleep apnea prior to the clinical trial; nor would they be expected to have had direct access to PAP therapy for their patients. Although screening for sleep apnea in cardiology centers is becoming increasingly common, adverse consequences of adaptive servo-ventilation in CSA patients with concomitant reduced ejection fraction heart failure [14] would tend to drive these patients toward non-PAP therapies (such as TPNS). Sensitivity analyses in our PAP-treated and PAP-naïve subgroups without significant cardiac dysfunction confirmed that improvements in sleep disordered breathing, sleep, and daytime outcomes were similar to those observed for the groups as a whole.

The polysomnographic and clinical responses to TPNS observed in both the PAP-naïve and PAP-treated groups are a direct consequence of diaphragmatic capture and respiratory entrainment [23], leading to stabilization of ventilation, oxygenation, and sleep [21]. Comparable reductions in sleep disordered breathing account for similar improvements in sleep architecture, daytime somnolence, and quality of life, regardless of referral source, baseline patient characteristics, and co-morbidities. These responses to TPNS stand in contrast to those observed in PAP-treated CSA patients, in whom high rates of PAP-intolerance leave a substantial proportion of treated patients without treatment or symptomatic benefit [18–20]. TPNS adoption appears to be considerably broader than PAP therapy, as evidenced by low discontinuation rates (<5%) and an expressed willingness to have repeated the implant procedure in both groups. These findings suggest substantially greater levels of TPNS tolerability and effectiveness than PAP therapy.

TPNS responses in the PAP-treated subgroup could have been related to prior responses to PAP therapy before patients were enrolled in the parent clinical trial. Unfortunately, PAP titration responses were not collected in the parent clinical trial, so we were unable to assess whether TPNS treatment success was related to PAP efficacy. Nevertheless, sleep studies at the time of enrollment in the parent TPNS trial demonstrated somewhat greater CSA severity in the PAP-treated than PAP-naïve subgroup, suggesting that the former group presented a greater TPNS treatment challenge. Despite modest differences in CSA severity between groups, TPNS led to marked decreases in AHI that were roughly proportional to CSA severity at enrollment in both groups (see Table 2). After 1 year of TPNS therapy, the central apnea index fell to negligible levels, accounting for parallel decreases in the total apnea-hypopnea index in both groups. These reductions in AHI were comparable to those reported with CPAP [10] and ASV [14], although the ASV cohort started and finished the trial with lower AHI (31 and 7 events/h, respectively) than the TPNS subgroups. In contrast, TPNS resulted in greater reductions in arousal index and ESS. These improvements in sleep quality and daytime somnolence may be best explained by greater levels of nightly adherence to TPNS than CPAP or ASV. Prospective studies examining the comparative effectiveness of TPNS and PAP therapy will be required to determine whether PAP therapeutic efficacy differentially impacts therapeutic responses to TPNS.

Several limitations should be considered when interpreting the current findings. First, they reflect a post hoc, exploratory subgroup analysis that might not predict therapeutic responses prospectively. Nonetheless, the subgroups’ responses are consistent with the responses observed in the parent pivotal trial, and suggest that TPNS can provide effective initial and salvage therapy for patients with moderate to severe CSA. Second, while head-to-head trials of PAP-based vs. TPNS therapy would allow direct comparisons of therapeutic modalities, these trials would of necessity be unblinded and would exclude patients with reduced left ventricular ejection fraction [14]. Other than ASV, PAP modalities are not specifically indicated to treat CSA, and trials of these therapies were generally compared to an untreated rather than an active comparator group. Third, we acknowledge that no data were collected to determine the reason(s) that patients stopped PAP therapy, since these were not the primary focus of the parent clinical trial from which the current sub-analysis was derived. Specifically, the parent protocol did not collect information on PAP treatment withdrawal date or treatment response. Lacking this information, we could not determine whether our subgroups differed in sleep apnea characteristics while on PAP therapy, or whether PAP was ineffective rather than simply poorly tolerated. Despite protocol-related constraints in collecting pre-baseline treatment records, we still found that clinically meaningful responses to TPNS occurred in both groups, suggesting TPNS to be effective, tolerable, and safe. Fourth, we recognize that we were not adequately powered to compare treatment responses across all polysomnographic and clinical symptom domains between groups, but instead note that clinically meaningful improvements of similar magnitude were consistently achieved in both the PAP-naïve and PAP-treated groups across both the 6 and 12 month time points after TPNS therapy activation. Further research is needed to examine effects of demographic/anthropometric parameters, co-morbidities, baseline sleep apnea characteristics, PAP treatment responses, and the time since withdrawal of PAP therapy on TPNS responses between the groups. Such work would ultimately allow us to optimize patient selection criteria, characterize titration responses, and address questions about comparative effectiveness and tolerability.

Our findings from this post hoc analysis indicate that TPNS improves CSA and its immediate nocturnal and daytime sequelae in patients with and without prior PAP therapy. Moreover, TPNS offers CSA patients a well-tolerated, efficacious therapeutic approach with potential for substantial clinical benefit over PAP-based therapy. TPNS can be considered a viable therapeutic option for patients with moderate to severe CSA who are either PAP-naïve or in whom PAP is poorly tolerated, ineffective, or contraindicated.

## Supplementary Information

ESM 1 (PDF 119 kb)
