# Supplementary material for: Transvenous phrenic nerve stimulation improves central sleep apnea, sleep quality, and quality of life regardless of prior positive airway pressure treatment
Source: Sleep Breath. 2021 Mar 20;25(4):2053–63. doi: 10.1007/s11325-021-02335-x (PMC8590675; doi:10.1007/s11325-021-02335-x)
Supplement: Supplementary file 1 — (PDF 119 kb) [file 11325_2021_2335_MOESM1_ESM.pdf]

Sleep and Breathing electronic supplement for:

Transvenous Phrenic Nerve Stimulation improves central sleep apnea, sleep quality and quality of life regardless of prior positive airway pressure-treatment

Alan R. Schwartz, MD[a,b]; Lee R. Goldberg, MD, MPH[a]; Scott McKane, MS[c]; Timothy I. Morgenthaler, MD[d]

[a]University of Pennsylvania Perelman School of Medicine; [b]Universidad Peruana Cayetano Heredia (Lima, Peru); [c]Respicardia, Inc., Minnetonka, Minnesota; [d]Mayo Clinic, Rochester, MN

Correspondence:

Alan R. Schwartz MD
Email: aschwar02@gmail.com

Table 1. Polysomnogram and Clinical Metrics - Prior PAP-treated

		6 Months of Therapy		12 Months of Therapy	
**Endpoint**	**Baseline (N=53)**	**Result (N=45)**	**Change from baseline**	**Result (N=41)**	**Change from baseline**
Apnea Hypopnea Index (events/hour)	52 [35, 63]	20 [10, 31]	-29 [-40, -11]<br><.001	16 [10, 32]	-30 [-45, -7]<br><.001
Central Apnea Index (events/hour)	30 [17, 47]	3 [1, 8]	-25 [-40, -12]<br><.001	2 [0, 6]	-24 [-48, -15]<br><.001
Obstructive Apnea Index (events/hour)	2 [0, 4]	3 [1, 5]	1 [-1, 4]<br>0.017	2 [1, 5]	0 [-1, 3]<br>0.076
Mixed Apnea Index (events/hour)	1 [0, 5]	0 [0, 1]	-1 [-5, 0]<br>0.002	0 [0, 1]	-1 [-5, -0]<br><.001
Hypopnea Index (events/hour)	12 [2, 19]	9 [4, 15]	0 [-9, 3]<br>0.621	10 [6, 19]	2 [-7, 7]<br>0.562
4% Oxygen Desaturation Index (events/hour)	45 [33, 61]	16 [8, 32]	-25 [-39, -12]<br><.001	16 [8, 27]	-26 [-42, -6]<br><.001
Arousal Index (events/hour)	39 [30, 54]	20 [12, 29]	-17 [-30, -9]<br><.001	18 [14, 26]	-18 [-32, -8]<br><.001
Percent of Sleep with O2 Saturation<90% (%)	12 [4, 27]	3 [0, 17]	-3 [-17, 1]<br>0.003	5 [1, 13]	-5 [-13, -0]<br><.001
Percent of Sleep in N1 (% of sleep)	33 [19, 42]	21 [13, 29]	-10 [-16, 1]<br><.001	21 [14, 27]	-7 [-17, 3]<br>0.023
Percent of Sleep in N2 (% of sleep)	51 [42, 58]	57 [50, 65]	5 [-1, 13]<br>0.001	58 [48, 65]	4 [-5, 12]<br>0.116
Percent of Sleep in N3 (% of sleep)	3 [0, 7]	2 [0, 7]	0 [-2, 2]<br>0.622	2 [0, 7]	0 [-1, 1]<br>0.784
Percent of Sleep in REM (% of sleep)	10 [5, 16]	15 [10, 21]	3 [-2, 6]<br>0.052	15 [6, 21]	4 [-5, 8]<br>0.251
Epworth Sleepiness Scale (points)	12 [7, 16]	7 [4, 11]	-3 [-6, 0]<br><.001	8 [5, 10]	-3 [-8, 0]<br><.001
Fatigue Severity Scale	5.2 [3.4, 6.2]<br>(n=53)	4.7 [3.2, 5.4]<br>(n=47)	-0.4 [-1.3, 0.4]<br>P=0.017	4.6 [3.3, 6.0]	-0.2 [-1.4, 0.6]<br>0.084
Median [Q1,Q3] / Nominal 2-sided p-value from Wilcoxon Signed-Rank test for change from baseline to visit.					

Table 2. Polysomnogram and Clinical Metrics - PAP-naive

		6 Months of Therapy		12 Months of Therapy	
**Endpoint**	**Baseline (N=93)**	**Result (N=81)**	**Change from baseline**	**Result (N=78)**	**Change from baseline**
Apnea Hypopnea Index (events/hour)	41 [31, 56]	20 [9, 35]	-18 [-35, -6]<br><.001	19 [9, 34]	-19 [-33, -6]<br><.001
Central Apnea Index (events/hour)	21 [12, 36]	1 [0, 5]	-17 [-27, -8]<br><.001	1 [0, 4]	-17 [-30, -10]<br><.001
Obstructive Apnea Index (events/hour)	2 [1, 4]	3 [1, 9]	1 [-1, 6]<br><.001	4 [1, 10]	2 [-1, 6]<br><.001
Mixed Apnea Index (events/hour)	1 [0, 4]	0 [0, 0]	-1 [-3, 0]<br><.001	0 [0, 1]	-1 [-3, 0]<br><.001
Hypopnea Index (events/hour)	12 [4, 20]	7 [2, 17]	-1 [-7, 5]<br>0.273	8 [4, 17]	-1 [-7, 7]<br>0.582
4% Oxygen Desaturation Index (events/hour)	35 [24, 51]	20 [7, 33]	-12 [-28, -2]<br><.001	17 [8, 29]	-15 [-29, -3]<br><.001
Arousal Index (events/hour)	40 [27, 57]	21 [15, 31]	-14 [-28, -4]<br><.001	22 [15, 35]	-12 [-33, -4]<br><.001
Percent of Sleep with O2 Saturation<90% (%)	7 [2, 19]	5 [1, 16]	-2 [-7, 2]<br>0.026	3 [1, 12]	-3 [-9, 1]<br><.001
Percent of Sleep in N1 (% of sleep)	35 [21, 49]	27 [18, 38]	-4 [-19, 5]<br>0.004	28 [19, 40]	-4 [-18, 4]<br>0.002
Percent of Sleep in N2 (% of sleep)	45 [40, 56]	51 [40, 64]	4 [-6, 16]<br>0.020	53 [44, 59]	4 [-3, 13]<br>0.005
Percent of Sleep in N3 (% of sleep)	2 [0, 10]	2 [0, 11]	0 [-3, 2]<br>0.272	1 [0, 5]	0 [-4, 1]<br>0.015
Percent of Sleep in REM (% of sleep)	10 [6, 16]	13 [9, 19]	1 [-3, 8]<br>0.003	13 [8, 21]	3 [-2, 11]<br><.001
Epworth Sleepiness Scale (points)	8 [5, 13]	6 [3, 9]	-2 [-6, 0]<br><.001	5 [3, 8]	-3 [-6, 0]<br><.001
Fatigue Severity Scale	4.4 [3.3, 5.7]<br>(n=93)	3.4 [2.3, 4.8]<br>(n=83)	-0.5 [-1.9, 0.2]<br>P<.001	3.4 [2.4, 5.1]	-0.5 [-1.6, 0.4]<br>0.003
Median [Q1,Q3] / Nominal 2-sided p-value from Wilcoxon Signed-Rank test for change from baseline to visit.					

Table 3. Patient Global Assessment

Endpoint (N=14)	Prior PAP-treated		PAP-naive	
	6 Months	12 Months	6 Months	12 Months
Any improvement in PGA (%)[a]	85% (40/47)	84% (36/43)	72% (60/83)	74% (60/81)

[a] Reported as percent (n/N).

PGA = Patient global assessment

Table 4. Comparison of Results from 6 Month Randomized Period of Trial

	Prior PAP-treated			PAP-naive		
Variable	TPNS Change from baseline (n=25)	Control Change from baseline (n=22)	Between group p-value	TPNS Change from baseline (n=36)	Control Change from baseline (n=51)	Between group p-value
Apnea hypopnea index (events/hour)	-27<br>-40, -12	1<br>-5, 16	<.001	-20<br>-37, -8	0<br>-10, 15	<.001
Central apnea index (events/hour)	-30<br>-45, -13	1<br>-10, 11	<.001	-17<br>-32, -12	-3<br>-12, 7	<.001
Arousal Index (events/hour)	-17<br>-28, -7	1<br>-6, 12	0.002	-20<br>-33, -8	-4<br>-20, 2	<.001
Percent of sleep with oxygen saturation<90% (%)	-3<br>-17, 2	2<br>-4, 6	0.020	-4<br>-9, 0	0<br>-8, 4	0.052
Percent of sleep in rapid eye movement (%)	1<br>-5, 5	-2<br>-7, 2	0.263	1<br>-3, 8	0<br>-4, 4	0.085
Epworth Sleepiness Scale (points)	-4<br>-7, 0	1<br>-1, 2	0.004	-2<br>-6, 0	0<br>-3, 3	0.002

Reported as median and interquartile range.

P-value from Mann-Whitney test for difference in change from baseline between groups (2-sided).

TPNS = Transvenous phrenic nerve stimulation
